# PERITONEAL ADHESIONS TYPE I, III AND TOTAL COLLAGEN ON POLYPROPYLENE AND COATED POLYPROPYLENE MESHES: EXPERIMENTAL STUDY IN RATS

**DOI:** 10.1590/0102-6720201700020001

**Published:** 2017

**Authors:** Lucas Félix ROSSI, Manoel Roberto Maciel TRINDADE, Armando José D`ACAMPORA, Luise MEURER

**Affiliations:** Center for Experimental Research, Hospital de Clínicas de Porto Alegre, Federal University of Rio Grande do Sul, Porto Alegre, RS, Brazil.

**Keywords:** Surgery, Animal model, Hernia, Surgical mesh, Tissue adhesions.

## Abstract

**Background:**

Hernia correction is a routinely performed treatment in surgical practice. The improvement of the operative technique and available materials certainly has been a great benefit to the quality of surgical results. The insertion of prostheses for hernia correction is well-founded in the literature, and has become the standard of treatment when this type of disease is discussed.

**Aim:**

To evaluate two available prostheses: the polypropylene and polypropylene coated ones in an experimental model*.*

**Methods:**

Seven prostheses of each kind were inserted into Wistar rats (*Ratus norvegicus albinus*) in the anterior abdominal wall of the animal in direct contact with the viscera. After 90 days follow-up were analyzed the intra-abdominal adhesions, and also performed immunohistochemical evaluation and videomorphometry of the total, type I and type III collagen. Histological analysis was also performed with hematoxylin-eosin to evaluate cell types present in each mesh.

**Results:**

At 90 days the adhesions were not different among the groups (p=0.335). Total collagen likewise was not statistically different (p=0.810). Statistically there was more type III collagen in the coated polypropylene group (p=0.039) while type I was not different among the prostheses (p=0.050). The lymphocytes were statistically more present in the polypropylene group (p=0.041).

**Conclusion:**

The coated prosthesis was not different from the polypropylene one regarding the adhesion. Total and type I collagen were not different among the groups, while type III collagen was more present on the coated mesh. There was a greater number of lymphocytes on the polypropylene mesh.

## RESUMO

**Racional:**

A correção herniária é tratamento realizado rotineiramente na prática cirúrgica. O aprimoramento da técnica operatória e dos materiais disponíveis trouxe grande benefício na qualidade dos resultados cirúrgicos. A inserção de próteses para correção herniária é bem embasada na literatura e tornou-se o padrão de tratamento.

**Objetivo:**

Avaliar em modelo experimental dois tipos de próteses diferentes, de polipropileno e polipropileno revestido.

**Métodos:**

Foram inseridas sete próteses de cada tipo em ratos Wistar (*Ratus norvegicus albinus*) na parede abdominal anterior do animal em contato direto com as vísceras. Após o seguimento de 90 dias analisaram-se as aderências intra-abdominais, bem como avaliação por imunoistoquímica e videomorfometria do colágeno total, tipo I e tipo III. Também, fez-se análise histológica com hematoxylina-eosina para avaliação dos tipos celulares presentes em cada tela.

**Resultados:**

Aos 90 dias as aderências não foram diferentes entre os grupos (p=0,335). O colágeno total igualmente não foi estatisticamente diferente (p=0,810). O colágeno tipo III foi estatisticamente maior no grupo polipropileno revestido (p=0,039) enquanto o tipo I não diferiu entre as próteses (p=0,050). Os linfócitos foram estatisticamente mais presentes no grupo polipropileno (p=0,041).

**Conclusão:**

A prótese revestida não foi diferente da de polipropileno na variável aderência. O colágeno total e tipo I não foram diferentes entre os grupos enquanto que o colágeno tipo III foi mais presente na tela revestida. O número de linfócitos foi maior na tela de polipropileno.

## INTRODUCTION

Incisional hernia (IH) repair has been a problem for surgeons ever since the beginning of abdominal surgery^1^ ^,^ ^25^. It is the protrusion of abdominal content through a weak point in the wall, constituted by a scar from the previous surgery. The synthesis of the abdominal wall in complex situations such as severe infections, large incisional hernias, massive loss of tissue, necrosis and tumors is a difficult problem for a general surgeon to solve, especially when they do not have sufficient autogenous tissue available for adequate primary closure^15^ ^,^ ^16^ ^,^ ^27^. Increase in the number of laparotomies under unfavorable conditions led to difficult, sometimes impossible situations of abdominal closure, which consequently increased the prevalence of IH. It is a major cause of morbidity among patients^3^ and interferes in quality of life and cosmetic results^4^.

The incidence of IH varies from 1-11%, with a great increase if the cavity is closed under tension^16^. It continues to occur even several years after the base intervention^8^ ^,^ ^21^.

A synthetic material, to be used as prosthesis, should follow a few basic principles: it should not change in the presence of tissue fluids; not produces inflammatory or foreign body type reaction; not be carcinogenic or allergenic. It should be also chemically inert, sterilizable, mechanically resistant and affordable. The polypropylene prosthesis is better for the surgical treatment of IH^27^ ^-^ ^29^. However, intra-abdominal placement causes a major formation of adhesions, and may result in serious complications such as intestinal obstruction and enterocutaneous fistulae^7^. In order to diminish the rate of complications and the possibility of direct contact between the mesh and the abdominal viscera, a mesh formed by polypropylene associated with a layer composed of regenerated oxidized cellulose was developed. The latter aims to reduce adhesion formation (Proceed® Ethicon Inc, Somerville, NJ, USA). Indeed, according to the manufacturer, meshes with biological barriers are associated with much less adhesion formation than those without this structure.

The choice of mesh for incisional hernia repair may be a dilemma, since nowadays various types are available^10^ ^,^ ^20^ ^,^ ^24^. Therefore, here was used a mesh already widely chosen in surgical practice, i.e. of pure polypropylene with a new available mesh whose characteristic is the association of materials seeking to reduce intraperitoneal adhesions. The compound mesh (Ethicon^®^) is to be implanted in deep layers of the abdomen, aiming at intra-abdominal pressure to keep it in position, without visceral adhesion when it is in a position very close to them. Good tissue growth and resistance, with low rates of adhesion, were found with them^14^.

Thus, this research intents to analyze the adhesions formation with the use of these two meshes.

## METHODS

The study was done at the Unit of Animal Experimentation and at the Unit of Experimental Pathology, Hospital de Clínicas de Porto Alegre, Federal University of Rio Grande do Sul, Porto Alegre, RS, Brazil. The research had fundings that came from the Fund of Incentive to Research and Events (FIPE) and the bioethical aspects were approved by the Committee of Ethics in the Use of Animals under number 110079.

Was used an experimental model in Wistar male rats (*Rattus norvegicus albinus*), weighing 303-368 g approximately, three months old and apparently healthy. During the entire study they were kept under adequate environmental conditions according to the animal bioethical standards. They were allocated by simple randomization into two groups of seven, as follows: 1) polypropylene group (GPP) with polypropylene mesh measuring 3 cm long by 2 cm wide (6 cm^2^) to close the defect caused in the abdominal wall, and 2) coated polypropylene group (GPPR) with low density polypropylene mesh associated with regenerated oxidized cellulose and polydiaxone. The same procedures were adopted in the polypropylene group using the aforementioned prosthesis, i.e. high density polypropylene prosthesis (Marlex^®^ - Bard, UK) and low density coated prosthesis (Proceed^®^ - Ethicon, USA)

### Surgical technique

Anesthesia was performed with an injection of a solution of ketamine hydrochloride (100 mg/kg) and 2% xylazine hydrochloride (10 mg/kg) at a 2:1 dilution, intraperitoneally. All principles of antisepsis were followed and no antibiotic was used at any time in the trial. The animals remained under spontaneous respiration throughout the surgical procedure. A 4 cm long median longitudinal incision was performed in the anterior abdominal wall. The hypodermis was dissected seeking to expose the anterior abdominal wall and enabling the creation of an incisional hernia model. A mold constituted by a segment of malleable plastic, 2 cm wide by 3 cm long and dyed with methylene blue was used to demarcate the resection. In this way a defect was created in the anterior abdominal wall and then filled with the prostheses that were being studied.

The implants were allocated in an intraperitoneal position in direct contact with the viscera. The borders of the prosthesis were fixed to the anterior abdominal wall muscles with polypropylene 4-0 suture.

**FIGURE 1:**
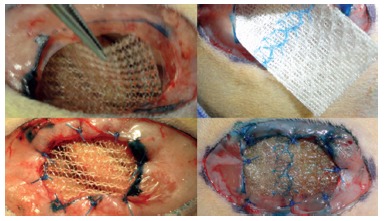
Insertion of prostheses, on the left the polypropylene mesh and its fixation with polypropylene 4-0 suture. On the right, mesh that separates tissues, fixed with the same suture.

In Figure 1 it can be seen the implantation, on the left, of the polypropylene mesh and on the right the coated one. The latter has different surfaces, and it is essential to be positioned correctly. The face that, theoretically, reduces adhesions is composed by regenerated oxidized cellulose and it was positioned in contact with the visceral content of the animal. The skin synthesis was performed with nylon 3-0 sutures.

### Observation period, death and analysis of variables

**FIGURE 2:**
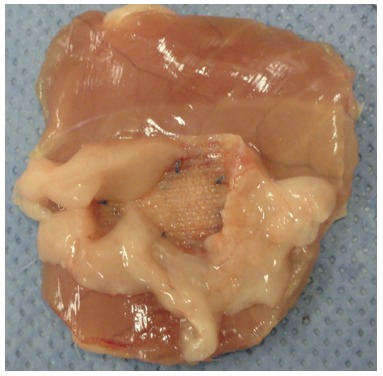
Resected anterior abdominal wall with the implanted prosthesis - polypropylene group

Immediately after the procedure, the animals remained in a heated incubator and were distributed into individual cages. The stipulated follow-up time was 90 days. After, the rats were killed in a CO^2^ chamber. The entire anterior abdominal wall with the implanted prosthesis was resected (Figure 2). The adhesions were graded using a specific score (Table 1). Afterwards they were placed in containers with a solution of 10% buffered formaldehyde, and identified individually according to group for later microscopic and immunohistochemical analysis.

**TABLE 1:** Adhesions score for evaluation

Parameter	Score				
	0	1	2	3	4
Adhesion % in each mesh	Absent	1-25% area of the mesh with adhesion	26-50% area of the mesh with adhesion	51-75% area of the mesh with adhesion	76-100% area of the mesh with adhesion
Adhesion thickness	Absent	< 5 mm	5-10 mm	11-15 mm	15 mm
Adhesion resistance	Absent	Spontaneous separation of the adhesion	Adhesion separated by traction	Adhesion separated by dissection	----

Values are reported as the sum of adhesions and intensity, ranging from 0 to 12

### Microscopic and immunohistochemistry

**FIGURE 3:**
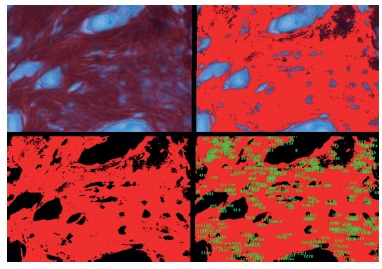
Process of videomorphometric analysis: collagen stained with picrosirius and quantitatively evaluated by number of pixels in the digitized image

The material was processed in the usual manner at the Laboratory of Experimental Pathology with the specimen processed in paraffin, cut at 4 μm and stained with H&E. The qualitative and quantitative analysis of collagen was done using an immunohistochemical technique applying type I and II anticollagen monoclonal antibody. The quantitative evaluation of total collagen was performed using the picrosirius technique. The analysis of all types of collagen was performed with computed optical videomorphometry measuring the number of pixels in each image (Figure 3). Pathologist who evaluated the microscopy did not know to which group each slide belonged, as blinded trial. In evaluating the H&E slides, the number of neutrophils, lymphocytes, giant cells and number of times macrophages were wrapped around each mesh filament were quantified. The cell types in 10 sample fields (200x magnification) were counted for each slide and then the mean values for each variable.

### Statistical analysis

 The data with a normal distribution were analyzed using the Student t test at a 95% level of confidence (p<0.05), with mean and standard deviation. The difference of the means (effect size - E/S) was done. The predictive variable of the study was the use of two types of surgical meshes, and the outcome variable was the quantification of adhesions, weight, cell types, total, type I and type III collagen.

## RESULTS

The baseline weight of the animals was not significantly different among the groups (p=0.965). Likewise, the difference in weight on the 90^th^ day between the polypropylene and coated polypropylene groups was not statistically different (p=0.241). (E/S -0.02 between the groups at the beginning of the study and E/S 0.76 between them at the end of the study).

### Macroscopic analysis

**FIGURE 4:**
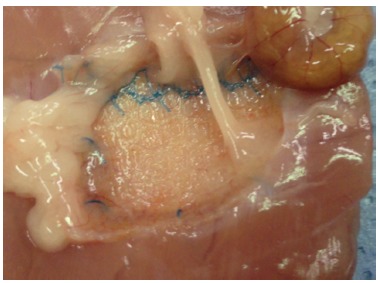
Colon and omentum adhesion in the Proceed group, notably more localized at the insertion of the mesh to the wall

Quantitative analysis showed that the mean score of adhesions of the GP was 7.5±1.65 and of the GPPR was 6.14±2.91 (Figure 4). Qualitatively, all animals in the polypropylene group presented adhesion of the omentum to the mesh. In the GPPR one animal presented adhesion to the colon (Figure 4), one of the small bowel and three in omentum. There was no statistical significance between the groups in the variable adhesion, measured by its own score and the effect size in favor of the polypropylene group was moderate (p=0.335 and E/S=0.56 respectively).

### Microscopic analysis

The mean number of neutrophils of the GP was 6.15 per field (SD=2.96) and 3.39 per field in the GPPR (SD=3.30) without a significant difference among them. (p=0.142). The mean number of lymphocytes in the GP was 4.65 (SD=1.46) and in the GPPR, 2.71 (SD=1.53). There was statistical significance in this variable (p=0.041). The mean of the giant cells in the GP was 3.1 (SD=0.87) and in the GPPR 4.02 (SD=1.78). There was no difference in this variable (p=0.260). The macrophages were evaluated according to the number of times they were wrapped around each filament of the mesh. In the GP the macrophages were wrapped around 3.06 (SD=0.56) times and in the GPPR 3.03 (SD=0.68) times. There was no statistical difference (p=0.940).

### Morphometric analysis: total, type I and type III collagen

The mean number of pixels, for the polypropylene group, counted automatically by specific software, was for total, type I and type III collagen respectively 1777.68 (SD=586.62); 321.68 (SD=121.80); 241.51 (SD=586.52). The coated group had 1889.77 (SD=1016.80); 201.91 (SD=72.55); 441.88 (SD=190.6) for the same sequence of variables cited. There was a statistical difference for the type III collagen variable (p=0.039), but none for type I collagen (p=0.050). Total collagen also did not present a statistical difference among the groups (p=0.810). As to effect size, we noted that type III collagen has a large effect size in the coated group, thus showing the intense expression of this type in that group.

**FIGURE 5:**
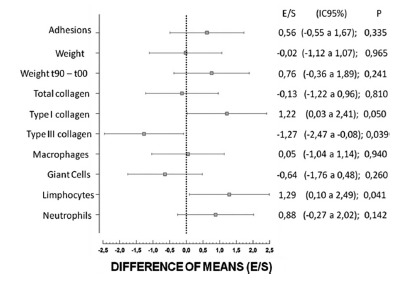
Forest graph showing the differences of standardized means (S/S), among the variable selected for the group treated with the coated prosthesis and the polypropylene one

The effect size (E/S) of each variable can be seen in Figure 5.

## DISCUSSION

The polypropylene prosthesis, experimentally described at the end of the 50s by Usher, has since then been considered very important to repair hernia defects in human beings^29^. The literature has a wealth of research on this type of prosthesis and its use is widely supported. The topic that must be dealt with is the development of intra-abdominal adhesions when this type of mesh is used. Postoperative adhesions have been reported as ranging from 20-50% for chronic pelvic pain to 74% for bowel obstruction. The use of barrier materials in an attempt to reduce adhesions has been constantly researched, but sometimes the results are contradictory^6^ ^,^ ^16^ ^,^ ^27^ ^,^ ^28^.

The main comparison in this study was to analyze a mesh that is widely used in surgery (polypropylene) with a coated prosthesis. The study follow-up time, 90 days, was established because there were no articles in the literature with prolonged follow-up. Because the coated prosthesis is a new therapy in the treatment of hernia, there are scarce studies on human beings, therefore the animal study model is appropriate for the objectives of the research study. Direct comparisons of non-absorbable materials to biologically absorbable materials in humans are scarce, and long term complications are uncertain^26^.

Technical information provided by the manufacturer of the coated prosthesis mention that it is composed of polypropylene, polydiaxone and a bioabsorbable anti-adhesion barrier compound called regenerated oxidized cellulose (Surgicel^®^). This latter layer has been used for the prevention of intra-abdominal adhesions and as a hemostatic barrier^2^. The polydiaxone polymer layer encapsulates the polypropylene layer to that of regenerated oxidized cellulose, joining them together. The polypropylene layer is a flexible low weight mesh which allows tissue growth through its pores and is responsible for prosthesis adhesion.

The model of the creation of incisional hernia proved effective and appropriate to the aims of the research, as previously studied^6^. Fixation of the mesh in close contact with the viscerae was of the utmost importance and proved adequate to analyze the adhesions. All of the animals had a satisfactory synthesis of the borders of the prosthesis to the anterior abdominal wall.

Analysis of the data showed that there was no statistically significant difference between the weights of the animals at the beginning of the study and a negligible size effect, thus showing the equanimity in the selection of the groups. Likewise, the weight at the end of the 90 days was not statistically different among the groups treated. All 13 animals had a weight increase during the ninety-day follow-up time, with a greater increase, evaluated by size effect in the polypropylene group at the end of the study.

Ideally planned with seven animals in each group and with a follow-up time that could lead to losses during the course of the study; however, ultimately we only had a single death and the remainder of the animals completed the study without any complications related to the prosthesis insertion. The death occurred in an animal in the polypropylene group, probably as a result of respiratory infection during the first month of follow-up.

In the forest graph of Figure 5, it can be observed all sizes of the effect supplying the dimension of the difference among the prostheses for each of the work variables. This graph, difference of means, explains the results of the study as a whole, and it is thus a panel of the research findings.

There has been constant research on adhesion by score and analysis of barrier materials in the surgical literature^5^ ^,^ ^6^ ^,^ ^17^. In this variable, adhesion, there was no statistical difference between the meshes and the size effect was small. It is essential to understand the concept of size effect, because with this we can see the real difference between the groups even if the null hypothesis has not been refuted for some variables. Size effect values of up to 0.20 are considered small, between 0.20 and 0.50 medium and larger than 0.80, big^18^. The magnitude of effect on the adhesion variable is small between the two prostheses, and this leads to the idea that the coated mesh does not diminish adhesions up to the ninetieth day postoperatively. The coated prosthesis is composed of polypropylene, polydiaxone and regenerated oxidized cellulose. The latter is a bioabsorbable compound that is absorbed within 28 days. Thus, by the end of the study, at 90 days, the material had been completely absorbed and could not be seen macroscopically.

What we can formulate is that absorption of the regenerated oxidized cellulose exposes the polypropylene layer to the abdominal visceral content and that this consequently led to the adhesions found. Another experimental study showed that regenerated oxidized cellulose caused adhesions in almost half of the animals in the study^2^.

The distribution of adhesions in the coated group occurred irregularly but intensively on the borders of the prosthesis that were in contact with the abdominal wall. The use of polypropylene suture thread to fixate the mesh to the wall may explain the adhesion that is located closer to the periphery of the mesh. In the polypropylene group the adhesions occurred in a more distributed form throughout the area of the mesh, similarly to other studies^5^ ^,^ ^17^. All the animals in the polypropylene group had omental adhesions, but also small intestine and colon. This finding is well described by many available studies^2^ ^,^ ^5^ ^,^ ^6^ ^,^ ^11^ ^,^ ^17^ ^,^ ^29^. The adhesions could be easily detached from the mesh in both groups. Adhesions of the non-absorbable meshes are known and several degrees of adhesion can be present, with the formation of foreign body reaction when this type of prosthesis is used. The adhesion formation process is complex and is basically started by the tissue injury process which breaks down the balance between coagulation and fibrinolysis. Fibrin deposition results in a matrix where the fibroblasts produce extracellular matrix. The end of the process generates various degrees of adhesion^26^. There are many attempts at inhibiting or minimizing this reaction, considering the morbidity involved in the adhesions. The bio-prostheses seek native tissue repair with less inflammatory activity and foreign body formation^26^. An experimental study in rabbits showed that up to 40% of the meshes structured with polypropylene, even coated with bio-absorbable materials, cause adhesions in an animal model^19^.

Collagen was analyzed with the help of histochemistry aggregated to the computed videomorphometry technology. This allowed a quantitative characterization of the collagen variable (total, type I and type III). The number of pixels in each photo supplied quantitative data, since it is known that this type of data is better than the qualitative one in statistics. For this purpose we measured the average number of pixels of four fields in each slide. This type of technique is well structured in the research group and publications used this methodology. Collagen research using the immunohistochemical methodology make the study reliable and its use is essential to identify collagens I and III^9^ ^,^ ^12^.

Type I collagen was not statistically significant among the groups with a large size effect (1.22) in favor of the polypropylene group. The argument concerning this is that type I collagen was found more in the polypropylene group than in the Proceed group even if there was no statistical significance.

Type III collagen was statistically significant among the groups and there was a large size effect (-1.27) in favor of the coated group. This means that type III collagen was expressed with a very large dimension in the coated group. The alterations in the metabolism of the collagen and the connective tissue contribute to hernia formation. Studies showed that patients with hernia present a larger amount of type III collagen. This type of collagen is considered immature, weak and favoring fragile fibers with a non-ideal quality found in the initial phases of healing^13^. In the present study, type III collagen was expressed more in the coated group and based on the result of the research this could increase hernia formation. Not only type III collagen expression is more intense in hernia patients, but also the reduction itself of collagen synthesis by fibroblasts^12^. Since it is a relatively new prosthesis, there are no data on in vivo research with a reasonable follow-up time to be able to associate these results with daily surgical practice.

Hence also total collagen was not statistically significant among the groups with an extremely low association force (-0.13). However, the analysis of type I and III collagen was essential for critical analysis of the prostheses. It is known that type I collagen is found in mature scar tissue and the reduction of the I:III collagen ratio is associated with hernia formation^13^. The incidence of incisional hernia and its recurrence are related to collagen metabolism. Metabolic causes are involved, as well as smoking, hormones and drug use. Hernia formation is influenced by alterations in the extracellular matrix and epidemiological studies show a defect in the biology of the scar tissue^22^.

In the histology of the prostheses, no difference was noted in macrophages, giant cells and neutrophils, and thus they are not significant. Only the lymphocytes presented a statistically significant difference. Since analysis was done in a single step at 90 days, we cannot infer anything about cellular behavior in more than one time step, as reported by others^30^. We know that the lymphocytes were the only cell type that showed a significant difference. These are involved in the non-specific inflammatory response, they recruit macrophages and influence phagocytic activity directly.

There is strong evidence, widely supported in the literature, regarding the use of prostheses. However, we do not believe that the selection of the type of prosthesis that can be used is a simple decision. Specific characteristics of the prosthesis and the patient should always be analyzed together for an appropriate choice. There are few clinical studies available with a coated prosthesis. One of the few available shows that, in videolaparoscopic surgery to correct ventral hernia, it is an adequate prosthesis with low rates of complication^23^.

What we presented in this study showed that in an experimental model the use of mesh with a barrier mechanism does not prevent the possibility of adhesions. Consequently all complications inherent to the process of adhesion formation, such as bowel obstruction, fistulae and granulomas may be found using this type of prosthesis. Indeed, as mentioned, there are no data from clinical trials with a long term follow-up using mesh with a layer of protection against adhesions. Further clinical trials will reveal, with a higher degree of scientific evidence, information about the behavior of individuals who were treated with this kind of mesh.

## CONCLUSION

Quantity of adhesions was not different for the polypropylene and coated polypropylene meshes. Type I collagen is more prominent on the polypropylene mesh (E/S). Type III collagen is more often present on the coated polypropylene mesh. The quantity of total collagen did not show any difference between the meshes. The number of lymphocytes is greater in the coated polypropylene group.
